# A Rare Case of Subcutaneous Scalp Mass As the Initial Presentation of Melanoma of Unknown Primary

**DOI:** 10.7759/cureus.89752

**Published:** 2025-08-10

**Authors:** Megan Miller, Greeshma Ragu, Patrick D Plummer, Hui Un Kim, Matthew Passeggiata, Shawn Clark

**Affiliations:** 1 Department of Surgery, Ross University School of Medicine, Bridgetown, BRB; 2 Department of Surgery, Western Reserve Health Education/Northeast Ohio Medical University (NEOMED), Warren, USA; 3 Department of Surgery, American University of Antigua, St. John's, ATG; 4 Department of Surgery, Western Reserve Hospital, Cuyahoga Falls, USA

**Keywords:** atypical melanoma, malignant melanoma, melanoma of unknown primary, melanoma surgery, metastatic melanoma

## Abstract

Melanoma is a form of skin cancer responsible for the majority of skin cancer-related deaths. Melanoma of unknown primary origin that presents as a periosteal scalp mass without overlying skin changes is rare and diagnostically challenging. Clinically, melanoma often presents as a skin lesion that is asymmetric, has irregular borders, exhibits multiple colors, is larger in size, and shows recent changes in appearance. The patient in this case is an 89-year-old male who initially presented with a subcutaneous cyst on the posterior scalp. Pathological examination of the excised mass ultimately confirmed malignant melanoma of unknown primary origin. A positron emission tomography scan performed after surgery revealed abnormal uptake in bilateral pulmonary nodules and multiple bilateral hilar lymph nodes. This case report highlights the clinical presentation, diagnostic challenges, and possible etiologies of melanoma with an unknown primary site that has metastasized to an unusual location.

## Introduction

Melanoma is a type of skin cancer that originates from melanocytes, the pigment-producing cells primarily located in the skin. Although melanoma accounts for only about 3% of all skin cancers, it is responsible for approximately 65% of skin cancer-related deaths. In the United States, melanoma is now the fifth most commonly diagnosed cancer [1]. According to the American Cancer Society, an estimated 104,960 new cases and 8,430 deaths from melanoma are expected [2]. Unlike other skin cancers, melanoma can also arise in non-cutaneous locations such as the mucosal surface, uvea, gastrointestinal tract, and brain, contributing to its high metastatic potential, with the ability to spread locally, regionally, and distantly [3].

When malignant melanoma is diagnosed, it is important to evaluate the patient’s family history, sun exposure, tanning bed use, and the presence of atypical mole syndromes. Individuals with fair skin, lighter phenotypic characteristics, lower socioeconomic status, and significant sun exposure are at increased risk of developing malignant melanoma. The pathophysiology of melanoma is primarily driven by ultraviolet-induced DNA mutations, which play a significant role in melanoma development and are associated with a high mutation burden. Genes associated with a predisposition to melanoma include *CDKN2A*, *CDK4*, and *MC1R *[3]. The risk of metastasis in malignant melanoma is closely associated with the depth of invasion and the presence of ulceration in the primary lesion. Early-stage melanoma progression involves local invasion, angiogenesis, extravasation, dissemination, and eventual colonization of distant organs [3,4].

## Case presentation

An 89-year-old male with a significant past medical history of prostate cancer and bladder cancer (transitional cell carcinoma), status post radical cystectomy with ileal diversion, presented with a subcutaneous scalp mass that had doubled in size over the past six months. The only symptom he reported was pressure-induced pain from the mass. The patient denied systemic symptoms such as fever or chills, night sweats, headache, unintended weight loss, changes in vision, and changes in hearing. He had a family history of prostate cancer in his brother and lymphoproliferative disorder in his sister. The patient was a lifetime non-smoker, does not consume alcohol, and denied recreational drug use. The patient reports extensive sun exposure in the past. Physical examination revealed a mass measuring about 3.5 x 3.5 cm, soft, pliable mass on the posterior scalp with no visible superficial lesions, ulceration, overlying erythema, lymphadenopathy, or pigmentation. There was low suspicion for this mass being malignant as there were no typical clinical features of melanoma in this patient. The differential diagnosis at that time was benign lipoma or cyst.

**Figure 1:**
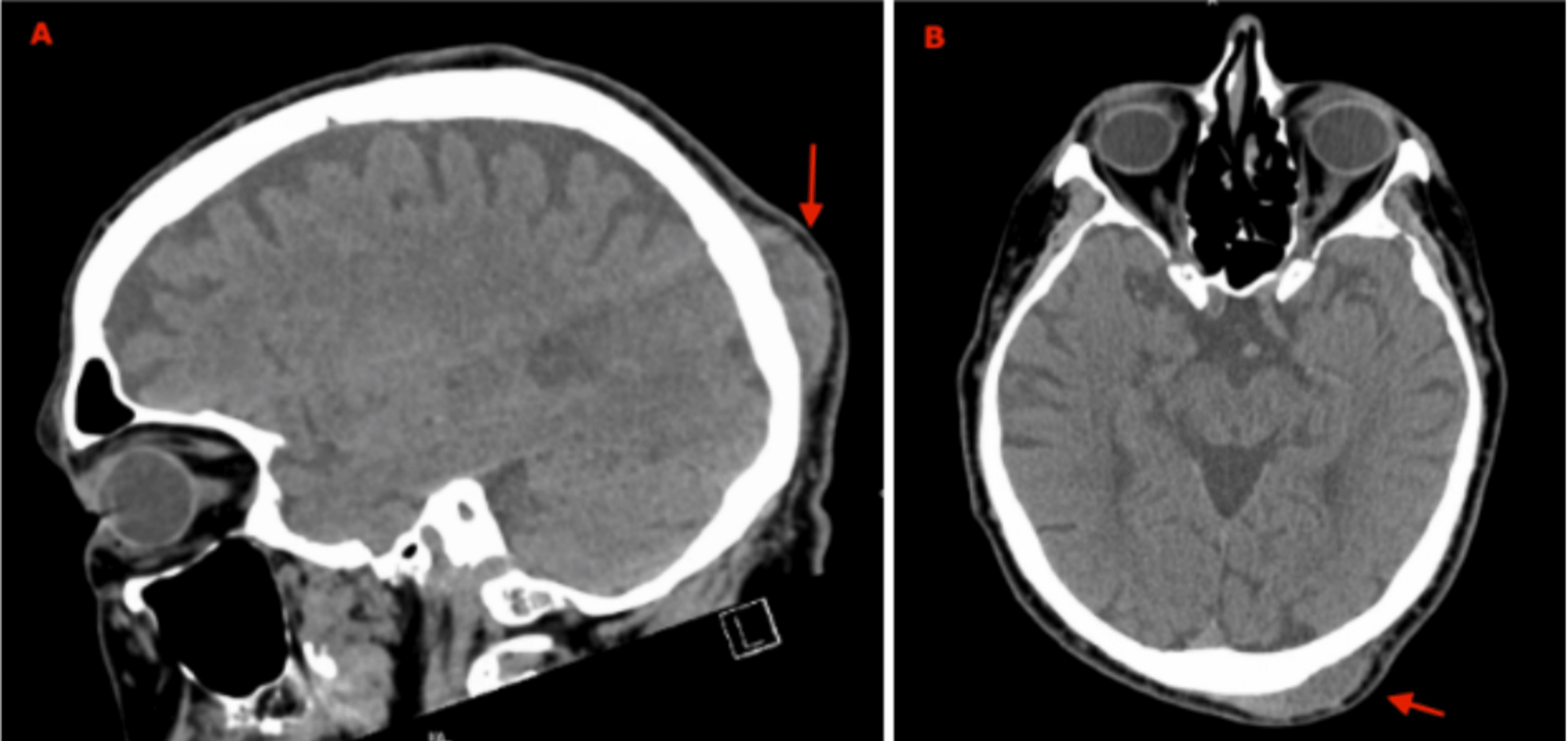
CT head showing 5 cm subcutaneous posterior left scalp mass abuts the calvarium with no underlying bony abnormality, periosteal reaction, or measles effect on the underlying brain parenchyma. A: Sagittal view of the head with the lesion (red arrow). B: Transverse view of the head with the mass (red arrow).

Preoperative head CT was obtained to assess the size and location of the mass (Figures 1A, 1B), which showed 5 cm subcutaneous left posterior scalp with no underlying bony abnormalities. The patient subsequently underwent soft tissue mass excision of the posterior scalp. Intraoperative findings showed scalp mass completely under the scalp and adherent to the periosteum of the skull. The dissected mass was a black gelatinous mass measuring 4.0 x 4.4 x 2.9 cm with irregular borders and was sent for pathology. The postoperative course was uneventful with minimal blood loss and no complications. The patient recovered well from the procedure with no clinical signs of recurrence on postoperative visits. Postoperative labs including lactate dehydrogenase (LDH) were all within normal limits.

**Figure 2:**
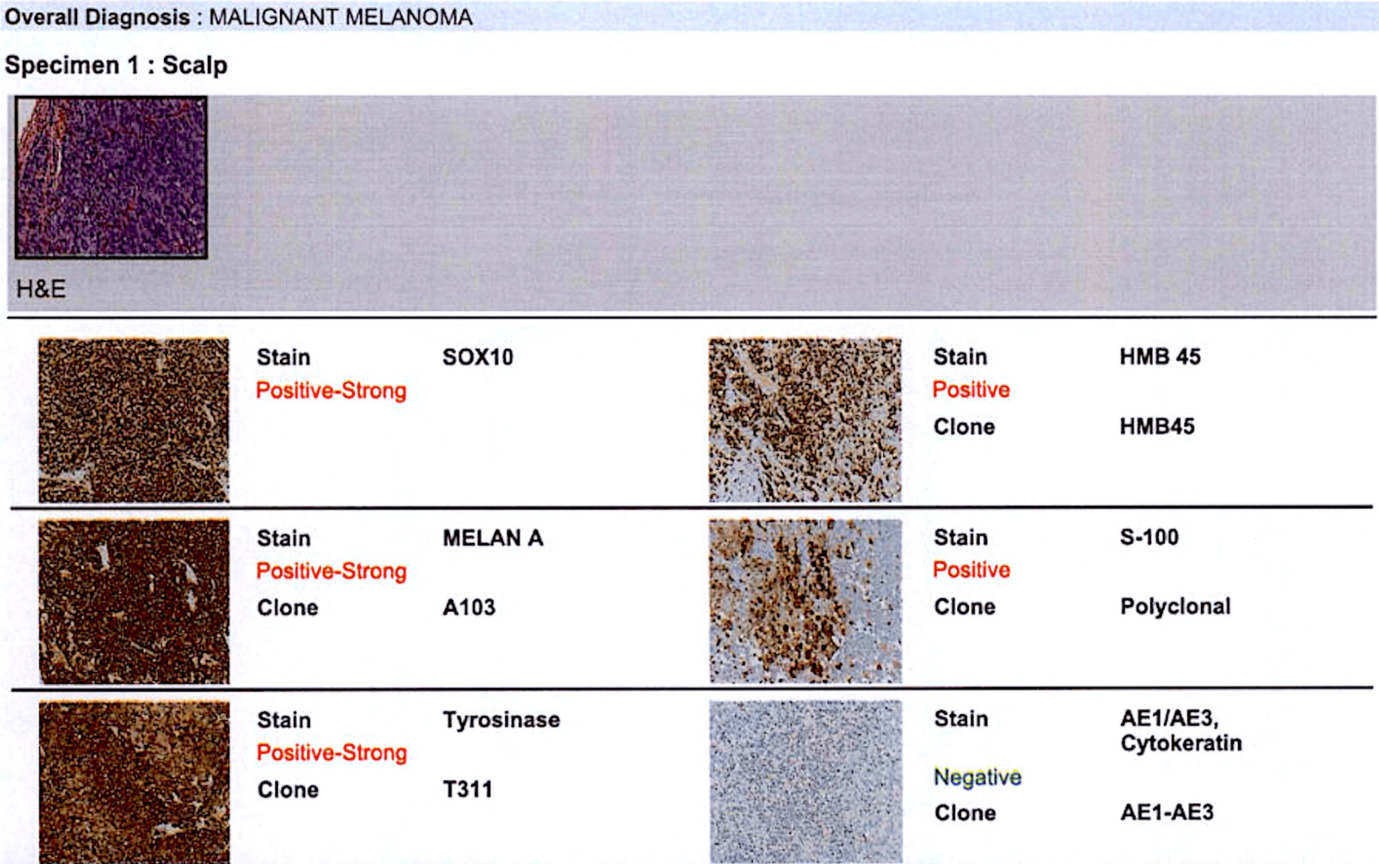
Histopathological analysis confirmed malignant melanoma, with tumor cells positive for SOX10, HMB-45, MELAN-A, S100, and tyrosinase, and negative for AE1/AE3.

Surprisingly, histopathological analysis revealed malignant melanoma, a diagnosis that was unexpected based on the clinical and radiographic presentation. The mass was positive for SOX10, HMB-45, MELAN-A, S100, and tyrosinase (Figure 2). It was negative for AE1/AE3. No cutaneous, mucosal, or ocular primary source was identified, consistent with melanoma of unknown primary (MUP). The patient had history of significant sun exposure and the absence of a pigmented skin lesion; spontaneous regression of a primary lesion was considered plausible. Molecular testing through next-generation sequencing (NGS) revealed no detectable mutations in *BRAF*, *NRAS*, or *KIT *genes. This NGS data was obtained for molecular profiling for prognostic information and for future therapeutic decisions.

**Figure 3:**
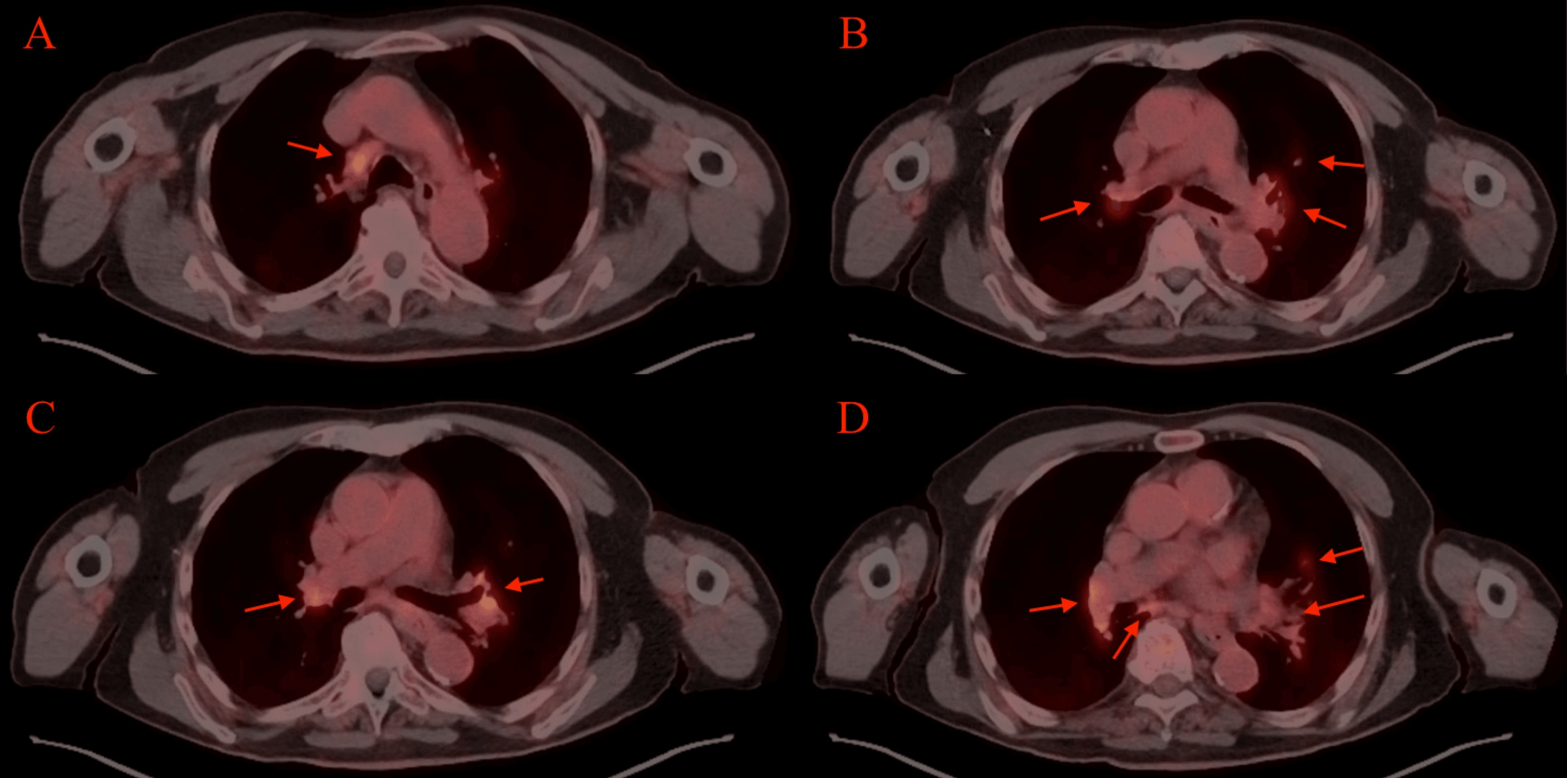
The PET scan demonstrated abnormal uptake in multiple bilateral pulmonary nodules (SUV 2.8), as well as in the superior mediastinal, pretracheal, subcarinal, and bilateral hilar lymph nodes (SUV 6.1). SUV: standardized uptake value, PET: positron emission tomography. SUV >2.5 often suggest malignancy. A-D represent sequential transverse PET scan sections from superior to inferior levels, respectively. A: Focal increased uptake in the right paratracheal or superior mediastinal lymph node region. B: Multiple bilateral increased uptake in the mediastinal, pretracheal, and hilar lymph node stations. C: Increased uptake is seen in bilateral hilar regions, consistent with lymphadenopathy or possible pulmonary nodules. D: Diffuse abnormal uptake persists in subcarinal and hilar nodes, with additional pulmonary involvement. Red arrows indicate areas of abnormal uptake.

A positron emission tomography (PET) scan from the skull to the thigh revealed abnormal uptake suspicious for metastasis in multiple bilateral pulmonary nodules and bilateral hilar lymph nodes (Figures 3A-3D). An attempt was made to obtain a CT-guided lung biopsy; however, the procedure was unsuccessful due to the patient's excessive and uncoordinated respiratory movements and inability to cooperate. The patient was counseled on the potential need for surgical re-excision of the posterior scalp to achieve negative margins, but he expressed his wish not to pursue any further surgical intervention. He was advised to follow up with oncology for evaluation for immunotherapy and palliative care options. General surgery follow-up was lost after this discussion.

## Discussion

Malignant melanoma most commonly arises from cutaneous lesions visible on clinical inspection, following the hallmark ABCDE criteria: asymmetry, border irregularity, color variation, diameter greater than 6 mm, and evolving morphology [3]. However, this case demonstrates a rare presentation of melanoma of unknown primary (MUP) manifesting as a deep, subcutaneous scalp mass without any overlying skin changes. Such cases challenge traditional diagnostic paradigms and highlight the importance of histopathologic assessment for atypical lesions.

MUP is defined as metastatic melanoma involving lymph nodes, subcutaneous tissue, or visceral organs without an identifiable primary lesion despite thorough evaluation of all cutaneous, mucosal, and ocular surfaces [4]. Spontaneous regression of a primary cutaneous melanoma is the most widely accepted explanation, which is plausible in our patient given his extensive sun-exposure history and the absence of a documented pigmented lesion. MUP accounts for only 2%-6% of all melanoma diagnoses [5]. A well-supported theory suggests that spontaneous regression of the primary lesion may occur without the patient’s awareness. Melanoma and melanoma of unknown primary share similar prognostic factors and survival rates are comparable [5]. A biopsy of any suspicious skin lesion is required for definitive diagnosis. For melanoma in situ, Mohs micrographic surgery may be considered when excisional biopsy reveals margins of 10 mm on the trunk and 12 mm on the face and neck [6]. If there is no evidence of metastasis, surgical removal of the melanoma followed by close monitoring is the standard treatment.

The scalp mass in this patient initially resembled a benign cyst or lipoma, lacking hallmark features such as pigmentation, ulceration, or induration. This diagnostic pitfall underscores the need for clinical vigilance, particularly in elderly patients or those with a prior cancer history. Histopathology ultimately confirmed malignant melanoma adherent to the periosteum, emphasizing that definitive diagnosis relies on tissue sampling rather than clinical impression alone. This case is strongly suggestive of MUP as no typical cutaneous primary melanoma was identified on both clinical exam and PET/CT scans.

Suliman et al. reported an atypical case of nodal melanosis coexisting with metastatic melanoma, fearsome prognosis of nodal metastasis [7]. Likewise, Huang et al. described an incidentally discovered rectal melanoma during hemorrhoidectomy, illustrating the importance of maintaining diagnostic vigilance for melanoma in unusual anatomical locations [8]. These two studies, along with this case of MUP, demonstrate that atypical demonstrations of melanoma can be easily overlooked, potentially progressing to nodal melanosis and resulting in poor prognosis.

The diagnostic workup of melanoma should be comprehensive and multidisciplinary. Full-body skin, scalp, ocular, mucosal, and nail inspection, ideally with dermoscopy, is critical [3]. PET/CT scan serves as the primary imaging modality for staging and detecting occult metastases, as demonstrated in our patient, and brain MRI is advisable for lesions near the scalp or calvarium given the risk of central nervous system (CNS) spread. Sentinel lymph node biopsy (SLNB) remains an important staging tool in melanoma ≥0.8 mm or with ulceration and can also aid prognostication in melanoma when technically feasible [3,4]. Molecular profiling through next-generation sequencing (NGS) is essential for therapeutic planning; in our patient, the tumor was negative for BRAF, NRAS, and KIT mutations. Gene expression assays, such as DecisionDx-Melanoma, may further refine recurrence risk stratification and guide follow-up strategies in cases where a primary site cannot be identified [3].

Management of malignant melanoma depends on stage and disease burden. Surgical excision with negative margins remains the primary treatment for localized lesions [3,9]. Systemic therapy is the standard for advanced or metastatic disease, with PD-1 inhibitors such as nivolumab or pembrolizumab as first-line agents with or without a CTLA-4 inhibitors, while BRAF/MEK inhibitors are reserved for mutation-positive cases. Radiotherapy may provide local control in select situations, particularly if re-excision is not feasible. Surveillance typically involves dermatologic examinations every three to six months for the first two years, with imaging guided by clinical stage and symptoms. Patients should also be counseled on sun protection and skin self-exams. Lifelong follow-up with dermatology, surgical oncology, and medical oncology is critical for optimizing outcomes [9].

## Conclusions

This case underscores several key clinical lessons. Clinicians should maintain a higher index of suspicion for melanoma in any atypical or enlarging soft tissue mass, even in the absence of visible skin changes. Histopathology must be obtained for definitive diagnosis, as imaging and clinical appearance can be misleading. Effective management requires interdisciplinary collaboration and increasingly relies on molecular diagnostics to guide treatment. Ultimately, our patient’s presentation exemplifies the diagnostic challenges of MUP, highlighting the importance of vigilance, tissue diagnosis, and individualized, multidisciplinary care.
